# Novel or No Novel?

**Published:** 1887-05-28

**Authors:** 


### NOVEL OR NO NOVEL?

We have received a mass of correspondence on this subject, which leaves no manner of doubt about the popularity of the novel. Eclipse is first and the rest nowhere. It is abundantly clear that, in the estimation of the majority of medical and semi-medical readers, a Scott is preferable to a Stuart Mill, whilst a Dickens leaves a Jeremy Taylor, or even a Trousseau, hopelessly in the rear. All men and women like to be amused and interested; it is only the few who have a real love of instruction for its own sake. The letters which have reached us are strikingly representative of all classes of our readers. As might have been expected, some of the younger nurses are enthusiastic in praise of the novel. It is the sweet champagne of the paper, and to them, at any rate, it makes the whole dinner delightful. One of them goes so far as to say, that "with the novel The Hospital is simply perfect." We are glad to hear it. "Give up the novel," says a medical student; "certainly not." This young gentleman looks forward to his weekly Hospital as a good "weekly boarder" anticipates the Sunday's plum-pudding at home. "Only a patient" reads the novel with the liveliest satisfaction. He is glad to see that doctors and nurses, the "human arbiters of our destiny," have a little of the common clay of humanity about them. He does not like the idea of their always living in the clouds. He thinks they will be much more serviceable to the patients if their hearts are not too tough to respond to the sentiments which are the common property of all mankind. The doctors, too—those withered and passionless anatomies who shake their heads so gravely, and look beyond their patients with sphinx-like eyes as if they searched the mysteries of a world unknown—even these men of buckram and infallibility have put in a dignified and solemn plea for the retention of the novel.

But stranger phenomenon still, and almost incredible, some of the matrons have let fall a gentle indication that perhaps the novel is not really a very bad thing after all. It is true they speak "with bated breath," though not with "whispering humbleness." It is evident that even in the heart of a hospital matron there is still a remembrance of the days when one footstep was wont to quicken the pulses and make the cheeks to glow: one voice was more musical than all other human speech; the clasp of one hand sent a thrill through the whole being such as no other experience of life was able to produce. It is even so: we are all human, or once were, and can no more uproot the memories of bygone years than heave a mountain from its base.

Why should we? It is not dreadfully wicked, or even reprehensible for a man to love a woman, or a woman a man. On the contrary, it is emphatically the very wine of life. The man or the woman who has never loved, has never known what it is truly to live. Undoubtedly it is "Better to have loved and lost, than never to have loved at all." We must then retain the novel. We can by no means find it in our hearts to be so cruel as to deprive youthful nurses, budding doctors, sentimental patients, portentous physicians and tender-hearted matrons of the weekly quarter of an hour of pleasant forgetfulness which the little story in The Hospital has manifestly given them.

No one, we think, really objects to a story. The objection, so far as there has been one, has been to the stage and the players. "Why lay the scene in a hospital," it is said, "and why make the players nurses and doctors?" Why, indeed? Except that the authors of the stories have apparently thought that to be the best way of interesting their readers. There is really no reason why the stories should be confined within such narrow limits; and we shall be glad to give our contributors the liberty of taking a bolder and more extended flight. We cannot promise that neither doctors nor nurses shall in future be introduced at all, but we are anxious that our more sober readers shall see nothing in our pages which can hinder the objects which the paper has in view. We are grateful for the criticisms which have reached us. Almost without exception they have been kindly and generous, and evidently dictated by the best of motives. They have shown us what a sincere interest our readers take in the paper and in hospital work; and we think we shall lose no editorial self-respect by saying that they have had, and will continue to have, that measure of consideration from us which honest and kindly intentions should always command. Another story will be commenced in an early number, and we venture to bespeak for it a fair and unprejudiced hearing.

